# Disulfide Bond Formation and ToxR Activity in *Vibrio cholerae*

**DOI:** 10.1371/journal.pone.0047756

**Published:** 2012-10-29

**Authors:** Vera H. I. Fengler, Eva C. Boritsch, Sarah Tutz, Andrea Seper, Hanna Ebner, Sandro Roier, Stefan Schild, Joachim Reidl

**Affiliations:** Institute of Molecular Biosciences, University of Graz, Humboldtstrasse, Graz, Austria; University of Osnabrueck, Germany

## Abstract

Virulence factor production in *Vibrio cholerae* is complex, with ToxRS being an important part of the regulatory cascade. Additionally, ToxR is the transcriptional regulator for the genes encoding the major outer membrane porins OmpU and OmpT. ToxR is a transmembrane protein and contains two cysteine residues in the periplasmic domain. This study addresses the influence of the thiol-disulfide oxidoreductase system DsbAB, ToxR cysteine residues and ToxR/ToxS interaction on ToxR activity. The results show that porin production correlates with ToxR intrachain disulfide bond formation, which depends on DsbAB. In contrast, formation of ToxR intrachain or interchain disulfide bonds is dispensable for virulence factor production and in vivo colonization. This study further reveals that in the absence of ToxS, ToxR interchain disulfide bond formation is facilitated, whereat cysteinyl dependent homo- and oligomerization of ToxR is suppressed if ToxS is coexpressed. In summary, new insights into gene regulation by ToxR are presented, demonstrating a mechanism by which ToxR activity is linked to a DsbAB dependent intrachain disulfide bond formation.

## Introduction

*Vibrio cholerae* is a Gram-negative, facultative anaerobic bacterium. It is the causative agent of cholera, which is endemic in India, Bangladesh, Southeast Asia, Africa and South America [1]. Infection starts with the oral ingestion of *V. cholerae* bacteria from the environment through contaminated food or water supplies [2], [3]. *V. cholerae* bacteria pass through the gastric acid compartment of the stomach, penetrate the mucus lining of the intestinal epithelia and start colonizing the small intestine. This compartment contains growth inhibitory substances, such as bile salts and organic acids and also factors of the innate immune system, e.g., complement secreted by intestinal epithelial cells [4] and defensins produced by Paneth cells [5]. Therefore, *V. cholerae* has developed the ability to survive, colonize and produce virulence factors [6] in spite of harsh stress conditions [7], [8].

Extensive studies of cholera pathogenesis revealed that production of the main virulence factors, namely cholera toxin (CT) and toxin-coregulated pili (TCP), is coordinated by a regulatory network [9]. This system is directly controlled by four transcriptional activator complexes identified so far, which act in a regulatory cascade and include AphAB, TcpPH, ToxRS and ToxT [10]–[14]. AphAB act at the beginning of the cascade and regulate transcription of the inner membrane located transcriptional regulator components TcpPH [14] and ToxRS [15]. ToxR is critical for regulation of virulence genes and together with TcpP, it activates transcription of *toxT* [12], [16]–[21]. Subsequently, the AraC-like transcriptional activator ToxT directly activates transcription of *ctx* and *tcp* loci, as well as additional genes [7], [19], [22]. *V. cholerae* strains lacking ToxT or ToxR do not produce CT or TCP and are avirulent [23]. Moreover, ToxR regulates the transcription of more than 150 additional genes [16], including *ompT* and *ompU*, which encode the outer membrane proteins OmpT and OmpU. Both porin genes are inversely regulated [24], [25], i.e., *ompU* transcription is activated, whereas *ompT* is repressed by ToxR as determined by OMP analysis and in vivo colonization [25], [26]. For the *toxT* promoter, ToxR and TcpP binding occurs such that ToxR binds on the distal end and acts as a “scaffold” protein by facilitating TcpP binding adjacent to the RNA polymerase binding site [27], [28]. Recently, it was shown that point mutations in the cytoplasmic domain of ToxR differentially activate *ompU* and *toxT* transcription. Based on this observation it was proposed that the orientation of ToxR on its corresponding operators differs for the *ompU* and *toxT* promoter regions [29].

The N-terminus of ToxR is located in the cytoplasm and contains the DNA-binding motif, followed by a transmembrane domain and then the periplasmic C-terminus [30]. A long lasting discussion exists about ToxR activity and ToxR dimer- and oligomerization. In *Escherichia coli*, ToxR-fusion proteins containing defined dimerization signals, were transcriptionally active. However, this activity was not conclusively confirmed in *V. cholerae* using the same or similar ToxR variants 30–34. As demonstrated by domain analysis, the presence of the ToxR transmembrane domain is essential for its activity [35], [36]. More recent evidence indicates that ToxR transmembrane segment itself possesses some function for ToxR activity and may be involved in bile dependent ToxR activation [37]. The ToxR periplasmic domain has been proposed to act as a sensor for environmental stimuli and contains two cysteine residues at amino acid position 236 and 293, which either can form homodimer or intrachain disulfide bonds [34]. The *toxS* gene is cotranscribed downstream of *toxR* [21]. ToxS is an inner membrane protein [7] and evidence suggests that ToxR and ToxS are interacting partners in the periplasm [38] and can form a heterodimer [34]. Additionally, knockout mutants in *toxS* negatively influence the transcriptional activity of ToxR [21], suggesting that ToxS facilitates the activity of ToxR or has implications for ToxR protein stability [38], [39]. However, major questions remain to be answered. What defines an active ToxR conformation? Is ToxR activity dependent on reduced or oxidized disulfide bonds? Is ToxR/ToxS interaction necessary to yield active ToxR complexes, e.g., homodimer, oligomer or heterodimer? And finally, does ToxR activity respond to environmental stimuli, cellular growth physiology and other input signals?

In this report, the molecular mechanisms that control activity of the membrane bound transcription factor ToxR were addressed using epidemic O1 El Tor and O395 classical strains. The study includes the interplay between ToxR and ToxS and the formation of ToxRS heterodimer. Furthermore, the redox state of ToxR cysteines were characterized in strains encoding knockout mutations in *dsbAB* and cysteine to serine substitutions in ToxR and ToxR activities were determined for virulence factor and porin expression.

## Materials and Methods

### Ethics statement

Mice were used for competition colonization experiments in strict accordance to the Guide for the Care and Use of Laboratory Animals of the National Institutes of Health, the national “Bundesgesetzblatt fuer die Republik Oesterreich”. Animal protocol (39/158 ex 2000/10), has been approved by the Austrian Federal Ministry of Science and Research Ref. II/10b and the Committee on the Ethics of Animal Experiments of the University of Graz. Housing of mice was conducted with food and water ad libitum and monitored in accordance with the rules of the Institute of Molecular Biosciences at the University of Graz.

### Bacterial strains, plasmids and culture conditions

Strains and plasmids used in this study are listed in Table 1. For construction of deletion and chromosomally encoded amino acid substitution mutations in *toxR*, the suicide vectors pCVD442 and pKEK229 were used. If not stated otherwise, *E. coli* and *V. cholerae* strains were transformed by electroporation. *E. coli* strain SM10λpir was used to introduce plasmids into *V. cholerae* by conjugation. *V. cholerae* P27459-S, a spontaneous streptomycin resistant mutant of *V. cholerae* O1 El Tor clinical isolate P27459 [40] or O395 [41], a spontaneous streptomycin resistant mutant of *V. cholerae* O1 classical clinical isolate were used as WT strains in all experiments. *E. coli* strains were grown using LB broth at 37°C. Unless stated otherwise, *V. cholerae* strains were grown using LB broth or minimal medium M9 supplemented with glycerol (0.4%) as a carbon source at 37°C. For optimal induction of virulence genes, *V. cholerae* O1 El Tor was grown using AKI conditions [42], whereas the *V. cholerae* O1 classical strain was grown in LB broth (pH 6.5) at 30°C [25]. If required, streptomycin, ampicillin and kanamycin were used at final concentrations of 100, 100, and 50 µg/ml, respectively. Other supplements were used in the following concentrations: IPTG (0.05 or 0.005 mM), glucose (0.2%), arabinose (0.2%) and sucrose (10%). Strains were stock frozen using LB medium containing glycerol (20%) at −80°C. If not noted otherwise, *E. coli* refers to XL1-Blue, *V. cholerae* to O1 El Tor isolate P27459-S and classical to classical O1 isolate O395.

**Table 1:** Bacteria strains and plasmids used in this study.

Strain or plasmid	Relevant characteristic	Reference
***E. coli*** ** strains**		
XL1-Blue	F′::Tn10 *proA* ^+^ *B* ^+^ *lac^q^* Δ(*lacZ*)M151 *recA1 endA1 gyrA46* (Nal^r^) *thi hsdR17* (r_K_^−^m_K_^+^) *supE44 relA1 lac*	NEB
DH5αλpir	F^−^ Φ80*dlacZ*Δ*M15*Δ(*argF lac*)*U169 deoR recA1 endA1 hsdR17* (r_K_^−^m_K_^+^) *supE44 thi-1 gyrA69 relA1 λ* recA::RPA-2-Te::Mu λ*pir*R6K, km^r^	[69]
SM10λpir	*thi thr leu tonA lacY supE recA*::RPA-2-Te::Mu λpirR6K, km^r^	[25]
***V. cholerae*** ** strains**		
O395	O1 Ogawa, classical, clinical isolate, India 1964, spontaneous sm^r^	[41]
P27459-S	O1 Inaba, El Tor, clinical isolate, Bangladesh 1976, spontaneous sm^r^	[40]
O395 Δ*dsbA*::km	*dsbA* replaced by km cassette, sm^r^, km^r^	This study
O395 Δ*dsbB*::km	*dsbB* replaced by km cassette, sm^r^, km^r^	This study
O395 Δ*toxR*	Deletion in *toxR*, sm^r^	This study
O395 Δ*toxRS*	Deletion in *toxR* and *toxS*, sm^r^	This study
P27459-S Δ*dsbA*::km	*dsbA* replaced by km cassette, sm^r^, km^r^	This study
P27459-S Δ*dsbA*::km Δ*toxRS*	*dsbA* replaced by km cassette, deletion of *toxR* and *toxS*, sm^r^, km^r^	This study
P27459-S Δ*dsbB*::km	*dsbB* replaced by km cassette, sm^r^, km^r^	This study
P27459-S *dsbC*::pGP	*dsbC* inserted by pGP704, am^r^	This study
P27459-S Δ*toxR*	Deletion in *toxR*, sm^r^	This study
P27459-S Δ*toxRS*	Deletion in *toxR* and *toxS*, sm^r^	This study
O395 Δ*toxR*::FLAG*toxR*	*toxR* replaced by FLAG*tox*R, sm^r^	This study
O395 Δ*toxR*::FLAG*toxR^CC^*	*toxR* replaced by FLAG*toxR^C236SC293S^*, sm^r^	This study
O395 Δ*toxR*::FLAG*toxR* Δ*toxS*	*toxR* replaced by FLAG*toxR*, deletion in *toxS*, sm^r^	This study
P27459-S Δ*toxR*::FLAG*toxR*	*toxR* replaced by FLAG*toxR*, sm^r^	This study
P27459-S Δ*toxR*::FLAG*toxR^CC^*	*toxR* replaced by FLAG*toxR^C236SC293S^*, sm^r^	This study
P27459-S Δ*toxR*::FLAG*toxR* Δ*lacZ*	*toxR* replaced by FLAG*toxR*, deletion in *lacZ*, sm^r^	This study
P27459-S Δ*toxR*::FLAG*toxR* Δ*toxS*	*toxR* replaced by FLAG*toxR*, deletion in *toxS*, sm^r^	This study
**Plasmids**		
pKEK229	Ori_R6K_ *mobRP4 sacB*, ap^r^	[70]
pGP704	Ori_R6K_ *mobRP4*, ap^r^	[25]
pCVD442	Ori_R6K_ *mobRP4 sacB*, ap^r^	[45]
pKan π	km^r^	[71]
pBAD18	Expression vector, arabinose inducible, ap^r^	[72]
pFLAG-MAC™	Expression vector with N-terminal FLAG-Tag, IPTG inducible, ap^r^	Sigma-Aldrich
pGPdsbC	pGP704 carrying internat fragment of *dsbC*′ ap^r^	This study
pKEK229dsbA::km	pCVD442 carrying up and down fragment of *dsbA* flanking a km cassette, ap^r^	This study
pKEK229dsbB::km	pCVD442 carrying up and down fragment of *dsbB* flanking a km cassette, ap^r^	This study
pCVD442lacZ	pCVD442 carrying up and down fragments of *lacZ*, ap^r^	This study
pCVD442toxR	pCVD442 carrying up and down fragment of *toxR*, ap^r^	This study
pCVD442toxRS	pCVD442 carrying up fragment of *toxR* and down fragments of *toxS*, ap^r^	This study
pdsbA	*dsbA* of P27459-S in pBAD18, ap^r^	This study
pdsbB	*dsbB* of P27459-S in pBAD18, ap^r^	This study
pFLAGtoxR	*toxR* of P27459-S in pFLAG-MAC™, ap^r^	This study
pFLAGtoxR^CC^	*toxR^C236SC293S^* point mutant of P27459-S in pFLAG-MAC™, ap^r^	This study
pFLAGtoxRS	*toxR* and *toxS* of P27459-P in pFLAG-MAC™, ap^r^	This study
pFLAGtoxR^CC^S	*toxR^C236SC293S^* point mutant and *toxS* of P27459-P in pFLAG-MAC™, ap^r^	This study
pFLAGtoxRS(Δ264)	pFLAGtoxRS carrying a 264 bp deletion in *toxS* generated by two internal *Acc*I sites, ap^r^	This study
pFLAGtoxRS_ompU	*toxR*, *toxS* and operator region of *ompU* of P27459-P in pFLAG-MAC™, ap^r^	This study
pFLAGtoxRS_toxT	*toxR*, *toxS* and operator region of *toxT* of P27459-P in pFLAG-MAC™, ap^r^	This study
pFLAGtoxRS_ompU(Δ264)	pFLAGtoxRS_ompU carrying a 264 bp deletion in *toxS* generated by two internal *Acc*I sites, ap^r^	This study
pFLAGtoxRS_toxT(Δ264)	pFLAGtoxRS_toxT carrying a 264 bp deletion in *toxS* generated by two internal *Acc*I sites, ap^r^	This study
pFLAGtoxR^CC^S_ompU	*toxR^C236SC293S^* point mutant, *toxS* and operator region of *ompU* of P27459-P in pFLAG-MAC™, ap^r^	This study
pFLAGtoxR^CC^S_toxT	*toxR^C236SC293S^* point mutant, *toxS* and operator region of *toxT* of P27459-P in pFLAG-MAC™, ap^r^	
pCVD442FLAGtoxR	pCVD442 carrying FLAG*toxR*, ap^r^	
pCVD442FLAGtoxR^CC^	pCVD442 carrying FLAG*toxR^C236SC293S^*, ap^r^	
pCVD442FLAGtoxRS	pCVD442 carrying FLAG*toxR* and *toxS*, ap^r^	

### Recombinant DNA techniques and construction of deletion mutants, point mutants and expression vectors

Oligonucleotide primers used in this study are listed in Table 2 and were purchased from Life Technologies (Life Technologies, Lofer). Chromosomal DNA was prepared as described previously by using ethanol salt precipitation [43]. Purification of DNA fragments from PCR samples and plasmid DNA preparations were performed using QIAgen QIAquick gel extraction, QIAquick PCR purification and QIAprep Spin Miniprep Kits according to the manufacturer's instructions (Quiagen, Hilden). Phusion high-fidelity polymerase (Finnzyme, Espoo) was used in PCR for DNA fragment generation for further subcloning and sequencing. For all other reactions, Taq DNA polymerase, restriction endonucleases and T4 DNA ligase were obtained from New England Biolabs (NEB, Ipswich). DNA sequencing was performed by the dideoxynucleotide chain termination method of Sanger et al. [44] with an automated DNA sequencer, performed at LGC Genomics, Berlin.

**Table 2:** Oligonucleotide primers.

Oligonucleotides	Sequence (5′ - 3′)^a^
SacI_dsbA_1	TTTGAGCTCCAAGAAGAGATCCCGATCGTCC
EcoRI_dsbA_2	TTTGAATTCCATGACTTTCTCCATTGGATTTATT
EcoRI_dsbA_3	AATGAATTCTAATCTCAACCCATGATTCGGTAT
XbaI_dsbA_4	TTTTCTAGAGATTAAACTGTTGCTGCCGTCAG
SacI_dsbB_1	TTTGAGCTCGTCTTCCTGCCAATGTT
EcoRI_dsbB_2	TTTGAATTCCACAGATAGATCCTTGTTAAAAAGA
EcoRI_dsbB_3	TTTGAATTCTAAGCCAATCGCATCGCTCAAT
XbaI_dsabB_4	TTTTCTAGATAGCATGGAGAGTGAGCCGCCACT
EcoRI_dsbC_1	ATTGAATTCGTGCAAACGTCTGGTGGT
XbaI_dsbC_2	ATTTCTAGAGAGCTCGTGACCCAGCAT
SacI_toxRS_1	TTTGAGCTCATTTGGAAATCACATCGCGCAAAC
BamHI_toxRS_2	TTTGGATCCTCCTAATCCGAACATCTAATGTCC
BamHI_toxR_3	TTTGGATCCAACCCTAACGATGCCATCAAAGT
BamHI_toxRS_3	TTTGGATCCTCCGATGACAATAGTGCAGAAAG
XbaI_toxRS_4	TTTTCTAGAATGACGTTTCCCCGCGGTGAG
XbaI_lacZ_1	TAATCTAGAACACATAACCCTGCAGTA
XhoI_lacZ_2	TTTCTCGAGCTCTACGGCGTACATCCCT
XhoI_lacZ_3	TTTCTCGAGTGCGTGTGGAATGTGACGAT
SacI_lacZ_4	ATGAGCTCTTATTGTGGGGATGACGCTTT
SacI_dsbA_5′	AATGAGCTCGCCACTTTACAAGAACCCCCG
XbaI_dsbA_3′	ATTTCTAGAGATTTACAAAGCCGATTAGCACTG
SacI_dsbB_5′	AATGAGCTCCAATTGAAACTGAAACTAATCCAAG
XbaI_dsbB_3′	AATTCTAGACTTTAAGCGCCTTTTTTATCAACC
HindIII_toxR_5′_FLAG	AATAAGCTTATGTTCGGATTAGGACACAACTCA
KpnI_toxR_3′_FLAG	AATGGTACCCTACTCACACACTTTGATGGCAT
KpnI_toxRC293S_3′_FLAG	AATGGTACCCTACTC**AGA**CACTTTGATGGCATCGTTA b
toxRC236S_5′	GGCTACCGTCAATCGAAC**TGA**GCGTTAAAAAATACAATGA b
toxRC236S_3′	TCATTGTATTTTTTAACGC**TCA**GTTCGATTGACGGTAGCC b
KpnI_toxRS_5′_FLAG	AATGGTACCCATGTTCGGATTAGGACACAACTCA
BglII_toxRS_3′_FLAG	TTAAGATCTTTAAGAATTACTGAACAGTACGGT
BamHI_ompU_5′	ATTGGATCCTCCTAAATCGGGTCGGGTT
BamHI_ompU_3′	AATGGATCCGGCTCAGCCATTTTCGTGGC
BamHI_toxT_5′	TTAGGATCCGTATAGCAAAGCATATTCAGAGA
BamHI_toxT_3′	ATTGTCGACTAAATAAACGCAGAGAGCCATC
c_FLAGtoxR_3′_F1	TGTCATCGTCGTCCTTGTAGTCCATCTAATGTCCCAGTATCTCCCTGT
c_FLAGtoxR_5′_F2	GGGACAGGGAGATACTGGGACATTAGATGGACTACAAGGACGACGATGA
c_FLAGtoxR_3′_F2	CTACTCACACACTTTGATGGCAT
c_FLAGtoxRC293S_3′_F2	CTACTC**AGA**CACTTTGATGGCAT b
c_FLAGtoxRtoxS_3′_F2	CTTTCTGCACTATTGTCATCGGTCTACTCACACACTTTGATGGCAT
c_FLAGtoxR_5′_F3	AACCAGTTAACGCTGAATTACATTC
c_FLAGtoxRC293S_5′_F3	GTTGCTAACCCTAACGATGCCATCAAAGTG**TCT**GAG b
c_FLAGtoxRtoxS_5′_F3	ATGCCATCAAAGTGTGTGAGTAGTCCGATGACAATAG
rpoB_fw^c^	CTGTCTCAAGCCGGTTACAA
rpoB_rv^c^	TTTCTACCAGTGCAGAGATGC
VC0633_fw	CTCGCGTACGTCTAAACTTCTTGG
VC0633_rv	CGGTTGTCTAGGCTGTTGTTAGAC
VC0984_fw	CCTTCATCAGCCACTGTAGTGAAC
VC0984_rv	GACCGCTATCAGAATAAGCAGTCG
VC1854_fw	ACCCACTAGTGATCGATGAAGACG
VC1854_rv	GCCATACTCAGCATATACACGAGC
VCr001_fw	AGGGAGGAAGGTGGTTAAGT
VCr001_rv	CGCTACACCTGAAATTCTACCC

a Restriction sites are underlined.

b Bold letters indicate codons changed to obtain desired amino acid mutations.

c Oligonucleotides for *rpoB* are according to reference [73].

Deletion mutations were generated as described by Donnenberg and Kaper [45] and represent start to stop codon deletions. DNA fragments of approximately 800 bp upstream and downstream of genes of interest were amplified by PCR (oligonucleotides labeled in the format X_Y, in which X stands for the restriction enzyme and Y for the respective gene) and digested with the corresponding restriction endonuclease. After ligation into suicide vector pCVD442 or pKEK229, derivatives were transformed into *E. coli* SM10λpir and were subsequently conjugated into *V. cholerae* [45]. Homologous integration of the plasmid into the chromosome, followed by negative selection for loss of plasmid in the presence of sucrose, allowed mutant strains to be constructed that have a deletion in the gene of interest. To improve selection of *dsb* deletion mutants, a km^r^ cassette was derived from pKanπ, Table 1, as an *Eco*RI fragment and ligated in between the *dsb* up and down flanking DNA fragments. The resulting *dsb* mutant strains carried chromosomal replacements of the *dsb* genes by km^r^ cassettes. The correct deletion for all mutants was confirmed by PCR (BioRad, Wien) (data not shown). Insertion mutants where constructed by using suicide plasmid pGP704 [25]. Therefore an approximately 500 bp internal fragment of the respective gene was amplified by using oligonucleotides labeled in the format X_Y, in which X stands for the restriction enzyme and Y for the respective gene, restricted with the corresponding endonucleases and ligated into similarly digested pGP704. Derivatives were transformed into *E. coli* SM10λpir and further conjugated into *V. cholerae*. Correct homologous integration of the plasmid into the chromosome was confirmed by PCR (BioRad, Wien) (data not shown) and maintenance of the plasmid was ensured by culturing the respective strains on media containing ampicillin.

For construction of expression plasmids, using pFLAG-MAC™ or pBAD18, the respective genes were amplified by PCR using oligonucleotides labeled in the format X_Y_5′ and X_Y_3′, in which X stands for the restriction enzyme and Y for the respective genes, Table 2. PCR fragments were digested with the respective restriction enzymes and ligated into similarly digested pFLAG-MAC™ or pBAD18. pFLAGtoxR was constructed by using oligonucleotides HindIII_toxR_5′_FLAG and KpnI_toxR_3′_FLAG. The C293S mutation in *toxR* was generated by using the oligonucleotide KpnI_toxRC293S_3′_ FLAG, which contains the required amino acid substitution for cysteine to serine residue. The C236S mutation was generated by SOE PCR (splicing by overlap extension), using pFLAGtoxR as a template and oligonucleotide pairs HindIII_toxR_5′_FLAG and toxRC236S_3′ as well as toxRC236S_5′ and KpnI_toxRC293S_3′_FLAG [46]. The two PCR products were used as the template in the second PCR with HindIII_toxR_5′_FLAG and KpnI_toxRC293S_3′_FLAG and the resulting PCR fragments were digested with *Hind*III and *Kpn*I and ligated into similarly digested pFLAG-MAC™. pFLAGtoxRS and pFLAGtoxR^CC^S were constructed by using P27459-S Δ*toxR*::FLAG*toxR* or P27459-S Δ*toxR*::FLAG*toxR* ^CC^ as templates and oligonucleotides KpnI_toxRS_5′_FLAG and BglII_toxRS_3′_FLAG. PCR fragments were digested with *Kpn*I and *Bgl*II and ligated into similar digested pFLAG-MAC™. The resulting plasmids were digested with *Bam*HI to integrate *ompU* or *toxT* operator sites. *ompU* and *toxT* operator fragments were amplified with oligonucleotides BamHI_ompU_5′ and BamHI_ompU_3′ or BamHI_toxT_5′ and BamHI_toxT_3′, respectively, and also digested with *Bam*HI. Similar constructs were digested with *Acc*I to generate a 264 bp deletion of *toxS*. Constructs were confirmed by PCR and DNA sequencing (data not shown).

Chromosomal FLAG-tagged *toxR* and amino acid substitution mutants were constructed by using SOE PCR. For amplification of PCR fragments, pFLAGtoxR and pFLAGtoxR^CC^ were used as templates. Oligonucleotides c_FLAGtoxR_5′_F2 and c_FLAGtoxR_3′_F2 respectively c_FLAGtoxRC293S_3′_F2 were used for generation for P27459-S Δ*toxR*::FLAG*toxR* and P27459-S Δ*toxR*::FLAG*toxR^CC^*. For construction of P27459-S Δ*toxR*::FLAG*toxR* Δ*toxS* oligonucleotides c_FLAGtoxR_5′_F2 and c_FLAGtoxRtoxS_3′_F2 were used. Fragments with about 800 bp each of flanking DNA regions of *toxR* with one end complementary to the first PCR fragment (see above) were amplified by PCR using oligonucleotides SacI_toxRS_1 and c_FLAGtoxR_3′_F1 and c_FLAGtoxR_5′_F3 or c_FLAGtoxRC293S_5′_F3 and XbaI_toxRS_4 for P27459-S Δ*toxR*::FLAG*toxR* and P27459-S Δ*toxR*::FLAG*toxR^CC^*, respectively. For construction of P27459-S Δ*toxR*::FLAG*toxR* Δ*toxS* oligonucleotides c_FLAGtoxR toxS_5′_F3 and XbaI_toxRS_4 were used. The three PCR products were used as templates in the second PCR with SacI_toxRS_1 and XbaI_toxRS_4 and the resulting PCR fragments were digested with *Sac*I and *Xba*I and ligated into pCVD442 that had been digested with same restriction enzymes. Resulting ligation products were transformed into *E. coli* SM10λpir and were further transferred into *V. cholerae* Δ*toxR* or Δ*toxRS* by conjugation. *V. cholerae* cells in which integration of the plasmid occurred by homologous recombination via one of the two fragments and a second homologous recombination step via the other fragment resulted in mutant strains harboring an integration of either FLAG-tagged *toxR* or FLAG-tagged *toxR* cysteine to serine substitution mutant in the *toxR* gene locus. The correct integration of all mutants was confirmed by PCR and DNA sequencing (data not shown).

### Membrane protein preparation

Proteins of the membrane and outer membrane of *V. cholerae* strains were prepared from cells either grown in LB medium or minimal medium M9 glycerol (0.4%). Cells were harvested by centrifugation, washed with HEPES pH 7.5 (10 mM) and lysed by sonification on ice according to standard protocols (Branson Sonifier 250A, Branson Ultrasonics Corp., Danbury). OMP preparations were performed as described previously [47]. For preparation of membrane proteins, lysed cells were centrifuged (13,000 g, 8 min, RT). The supernatants were then transferred to a new tube and centrifuged again (20,000 g, 30 min, RT). The membrane pellets were resuspended in HEPES pH 7.5 (10 mM) supplemented with protease inhibitor (Complete™, Boehringer Mannheim) to obtain proteins of the inner and outer membrane. In order to separate outer membrane proteins, the pellets were resuspended in HEPES pH 7.5 (10 mM) with sarcosyl (1%) and incubated for 30 min at RT. The suspensions were centrifuged (20,000 g, 30 min, RT) and the pellets, containing outer membrane proteins, were first washed and then resuspended in HEPES pH 7.5 (10 mM) supplemented with protease inhibitor. Protein amounts were determined by using the UV absorption 260/280 nm protocol according to Warburg and Christian [48].

### SDS-PAGE and immunoblot analyses

For whole cell extracts, *E. coli* or *V. cholerae* cultures were either grown in LB, induced with IPTG (0.005 to 0.05 mM) for one to two hours or in M9 glycerol (0.4%) minimal media and induced with IPTG (0.005 to 0.05 mM) for 6.5 h. Equal amounts of cells were harvested by centrifugation in an Eppendorf centrifuge. For immunoblot analyses of whole cell extracts the overall protein contents were assessed to contain similar protein levels by SDS-PAGE coomassie blue staining. Cell pellets were washed with media, resuspended in sample buffer either with or without the reducing agent β-mercaptoethanol and boiled for 10 min. OMP preparations and whole cell extracts were then separated by SDS-PAGE in polyacrylamide (15%) gels, using Mini-PROTEAN Tetra cell (Bio-Rad, Vienna). For detection of membrane and outer membrane proteins, equal protein amounts (60 µg and 4 µg, respectively) were loaded. After SDS-PAGE, proteins were either stained with Coomassie brilliant blue as previously described [49] or transferred for immunoblot analysis to a nitrocellulose membrane (Amersham-Bioscience, Freiburg). Immunoblot analyses were performed as described previously [50]. After transfer and blocking, the membrane was incubated for 2 h at RT with the primary antibody, mouse monoclonal anti-FLAG M2 antibody (Sigma-Aldrich, Taufkirchen) or anti-DDK monoclonal antibody (OriGene Technologies, Inc., Rockville) diluted 1∶2,000 in skim milk (10%) in TBS. The membrane was washed twice in TBS-TT (Tris-HCl pH 7.5, 20 mM, NaCl, 500 mM, Tween 20, 0.05%, Triton X-100, 0.2%) and once in TBS for 10 min each. The membrane was incubated with secondary antibody (horseradish peroxidase-conjugated goat anti-mouse, Dianova GmbH, Hamburg), diluted 1∶10,000 in skim milk (10%) in TBS, for 1 h at RT. Subsequently, the nitrocellulose membrane was washed three times in TBS-TT and once in TBS for 10 min each. Chemiluminescent detection was performed using the Immun-Star™ WesternC™ Kit (Bio-Rad, Vienna) and the result visualized using a Molecular Imager ChemiDoc™ XRS System (Bio-Rad, Vienna).

### qRT-PCR analyses

Primers used for quantitative reverse transcriptase PCR (qRT-PCR) are listed in Table 2 (labeled x-fw and x-rv, in which x stands for the respective gene and fw and rv for forward and reverse primers). The efficiencies of fluorescence signaling of the primer pairs amplifying the target and reference genes used in this study are at least 80% compared to a complete duplication per cycle and qRT-PCR was performed as described previously [51]. Briefly, RNA was isolated from six independent cultures grown in M9 glycerol (0.4%) medium at defined time points by using peqGOLD Bacterial RNA Kit (Peqlab, Erlangen). To remove chromosomal DNA, RNA was treated with RQ1 RNase-Free DNase (Promega, Mannheim). By using an iScriptTM Select cDNA Synthesis Kit (Bio-Rad, Vienna) cDNA was synthesized from 200 ng RNA, including controls lacking reverse transcriptase. Quantification of cDNA was performed with SYBR GreenER™ qPCR SuperMix for ABI PRISM® instrument (Invitrogen, Lofer), utilizing a Rotor-GeneTM 600 and Rotor-GeneTM 600 Series Software 1.7 (GenXpress, Wiener-Neudorf). Each reaction mixture contained primers (400 nM) and template (10 ng). Each independent sample was tested in triplicate. For each sample, the mean cycle threshold of the test transcript was normalized to that of reference gene *rpoB* [51] or 16 s rRNA and to one randomly selected *rpoB* or 16 s rRNA reference sample of the WT. Values above or below 1 indicate that the transcript is present in higher or lower numbers, respectively, in the mutant compared to the WT strain.

### CTX-kmΦ transduction

TCP production was determined by phage transduction frequency, utilizing phage CTX-kmΦ and TCP producing cells [52]. In short, lysogenic strain O395 CTX-kmΦ was grown over night in LB broth. Cells were centrifuged and the supernatant, containing CTX-kmΦ, was sterilized by filtration through a Whatman Klari-Flex filter unit with pore size of 0.22 µm (GE Healthcare, Vienna). Aliquots were stored at 4°C. For induction of TCP production, *V. cholerae* O1 El Tor was grown in AKI and *V. cholerae* O1 classical in LB broth (pH 6.5) at 30°C. Cells were incubated with CTX-kmΦ lysate for 30 min and subsequently plated in parallel on LB plates with sm and sm/km to determine transduction frequency.

### CT ELISA

CT production in culture supernatants was determined by the ganglioside G_M1_ ELISA [53]. *V. cholerae* O1 El Tor was grown in AKI and *V. cholerae* O1 classical in LB broth (pH 6.5) at 30°C to induce CT production. Cells were removed from CT containing supernatants by centrifugation and supernatants were stored at −20°C. ELISA plates (BD Falcon, Heidelberg) were coated with G_M1_ ganglioside (10 µg/ml) in Na_2_CO_3_ (10 mM) (Sigma-Aldrich, Taufkirchen) for 4 h at 37°C and washed four times with PBS-T pH 7.4 consisting of NaCl (137 mM), KCl (2.7 mM), Na_2_HPO_4_×2 H_2_O (8.1 mM), KH_2_PO_4_ (1.76 mM), Tween-20 (0.05%). Free binding sites were blocked with BSA (4 mg/ml) for 1 h at RT. After washing as described above, CT containing supernatants were diluted in PBS and added to the plate. Additionally, purified CT in PBS (Sigma-Aldrich, Taufkirchen) was inoculated in separate wells to generate a standard curve. ELISA plates were incubated with supernatants and purified CT for 1 h at RT and were washed again as described above. After incubation with the primary antibody (anti-CT antibody produced in rabbit, Sigma Aldrich, Taufkirchen), diluted 1∶2,000 in PBS containing BSA (4 mg/ml) for 1 h at RT, ELISA plates were washed four times with PBS-T. After incubation with the secondary antibody (goat anti-rabbit IgG - horseradish peroxidase, Amersham-Biosiences, Freiburg), diluted 1∶5,000 in PBS containing BSA (4 mg/ml) for 1 h at RT and subsequent washing, the ELISA plates were incubated with TMB Substrate Reagent Set (BioLegend, Vienna) for detection of CT. The reaction was stopped, by adding H_3_PO_4_ (1 M) and ELISA plates were measured at OD_450_ by using a microplate reader (FLUOstar Omega, BMG LABtech, Vienna).

### In vivo colonization studies

Competition assays for intestinal colonization in infant C57Bl/6 mice (Harlan Laboratories, Inc., Udine) (in vivo) and for growth in LB broth (in vitro) were performed as previously described [54], with a mixture of mutant (LacZ^−^) and isogenic WT (LacZ^+^) strains. The competitive index (CI) is the ratio calculated of CFU of mutant to WT recovered at 24 h, normalized to the input ratio.

### Statistical analyses

For data analyses Mann-Whitney U test, Kruskal-Wallis followed by Dunns test of selected pairs of columns or unpaired t test were used. Differences were considered significant for *P* values of <0.05.

## Results

### *dsbAB* mutants and porin production

**Figure 1:**
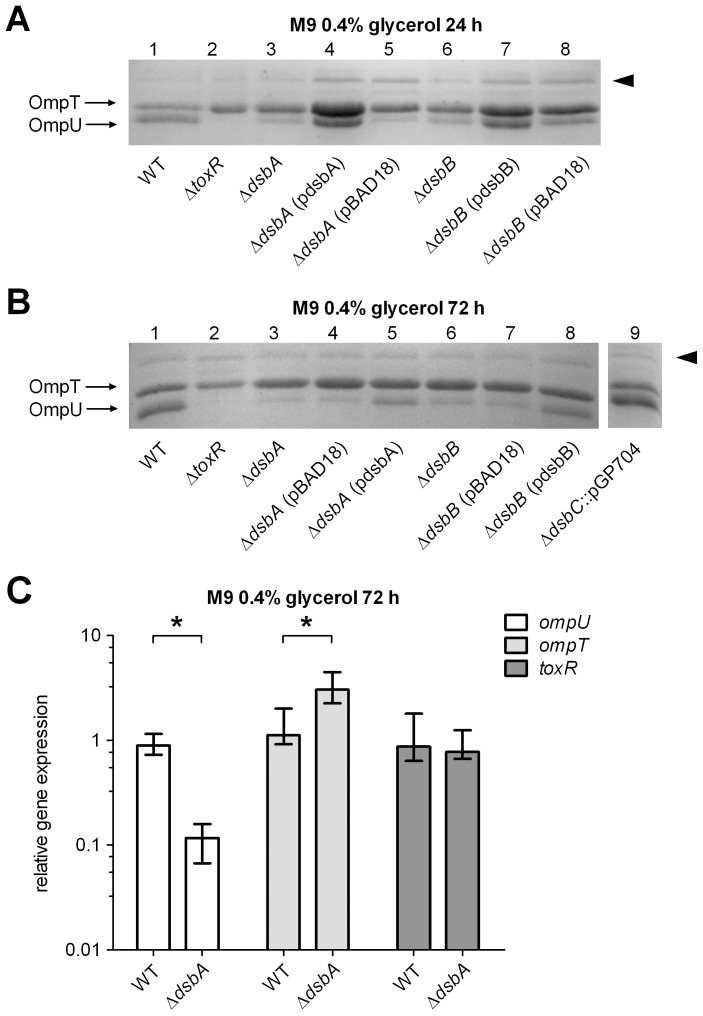
*dsb* knockout mutations and porin production in *V. cholerae* P27459-S. Panel A, B shown are OMP profiles on SDS-PAGE of WT, Δ*toxR*, Δ*dsbA*, Δ*dsbA* (pBAD18), Δ*dsbA* (pdsbA), Δ*dsbB*, Δ*dsbB* (pBAD18), Δ*dsbB* (pdsbB) and *dsbC*::pGP704 (only panel B) strains derived from cells grown for 24 h and 72 h in M9 glycerol, respectively. Arrows mark OmpU and OmpT. As a negative control, Δ*toxR* mutant strain showed no production of OmpU and derepressed OmpT protein level. The arrowhead on the right indicates a ToxR independent protein band used as loading control. Panel C, shown are qRT-PCR analyses of WT and Δ*dsbA* strain for *ompU*, *ompT* and *toxR* transcripts. Fold change ratios were calculated by comparing cDNA levels of genes of interest and the reference gene *rpoB*, derived from cells grown in M9 glycerol for 72 h. Data are presented as median fold change and the error bars indicate the interquartile range of each data set. Experiments were performed with at least six independent samples, utilizing the Mann-Whitney U test, *P*<0.05.

ToxR contains two cysteine residues in its periplasmic domain at amino acid position 236 and 293 [30]. Inter- as well as intrachain disulfide bond formation was found to exist within ToxR, which may alter ToxR activity [34]. To address whether ToxR activity depends on disulfide bond formation via the Dsb system [55], porin production was monitored by comparing a *V. cholerae* WT strain and corresponding *dsbA*, *dsbB* and *dsbC* deletion mutants. WT and the *dsbA* mutant showed similar growth kinetics under the conditions tested (Fig. S1). It is essential to monitor *dsb* defects during growth of cells in minimal media, because in rich LB broth small organic molecules are present that act as oxidizing agents and hence replace Dsb function [56]. Also for monitoring Dsb activity it might be of advantage to use C-sources, which are used for respiration rather than fermentation to establish ubiquinone depending e-transfer activating DsbB. Therefore, cultures were grown in M9 glycerol medium with high respiration to either stationary (24 h) or late growth phase (72 h) and then subjected to OMP preparation. As shown for O1 El Tor (Fig. 1A, B) and classical strain O395 (Fig. S2A), OmpU production was significantly decreased in samples derived from *dsbA* and *dsbB*, but not in samples derived from *dsbC* mutants (Fig. 1A). Complementing activity was observed by expressing *dsbA* or *dsbB* from pBAD18 in corresponding *dsb* knockout mutant strains (Fig. 1A, B). Importantly, if cells were grown in LB broth or M9 supplemented with glucose, no significant change in porin production was observed, neither in *dsbA* nor *dsbB* mutant strains (data not shown).

Furthermore, qRT-PCR was performed for *toxR*, *ompU* and *ompT* transcription in WT and *dsbA* mutant strains derived from late stationary grown cells (72 h) in M9 glycerol medium (Fig. 1B). In accordance with the OMP profile, the qRT-PCR data of WT compared with a *dsbA* mutant showed a 8-fold reduced *ompU* transcription, whereas *ompT* transcription was 3-fold upregulated. Notably, no alteration was observed for *toxR* transcription, indicating that a *dsbA* deletion has no effect on *toxR* transcription under the conditions tested.

### ToxR disulfide bond formation depends on ToxS and DsbA

**Figure 2:**
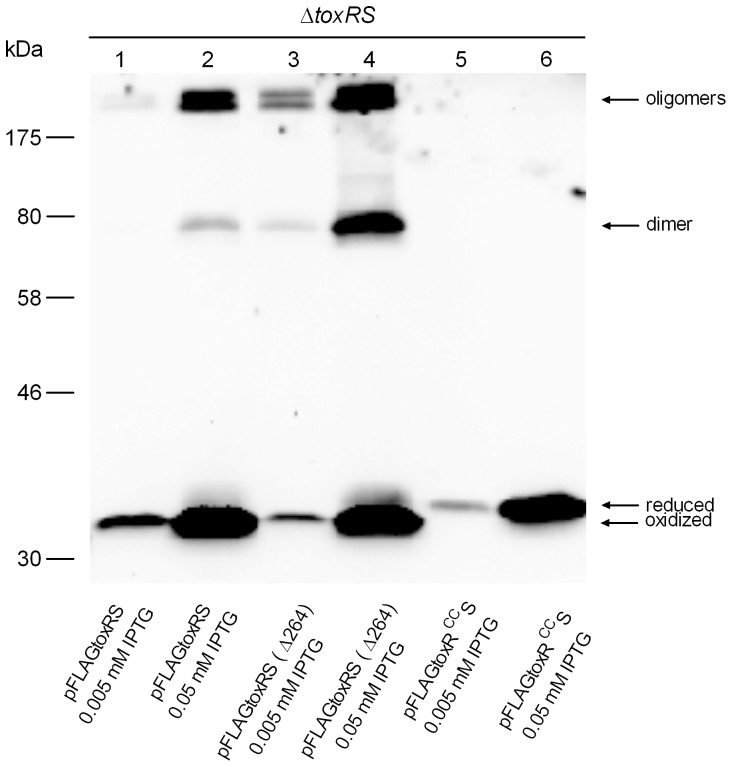
*toxRS* coexpression in *V. cholerae* P27459-S Δ*toxRS* mutant strain acts negatively on ToxR disulfide bond homodimer and oligomers. Shown is an immunoblot analysis derived from SDS-PAGE analysis performed under non-reducing conditions, utilizing anti-FLAG antibodies and *V. cholerae* cells harboring various pFLAGtoxRS expressing plasmids, grown in LB medium to mid-log phase and induced with IPTG. Molecular markers are indicated on the left side. Two different IPTG concentrations are indicated, showing different ToxR levels. Immunoblot analysis was performed at least three times, and results were reproducible.

In order to monitor ToxR protein in *V. cholerae*, *toxRS* was amplified using chromosomal DNA as template and *toxRS* encoding reporter plasmids were constructed. They additionally contained a DNA sequence encoding a FLAG tag peptide (N-DYKDDDDK-C) fused in frame to the *toxR* 5′ end, termed as FLAG-tagged *toxRS*. Cysteine residue 236 in ToxR was shown to contribute to ToxR intrachain disulfide bond formation [34]. Hence, the two periplasmic cysteine residues 236 and 293 were replaced with serine residues to construct a *toxR^CC^S* mutant. Furthermore, to address ToxS function on ToxR disulfide bond formation a control plasmid pFLAGtoxRS(Δ264) was constructed, harboring a 264 bp internal deletion in *toxS*, yielding an incomplete ToxS protein. Immunoblot analysis was performed using specific FLAG-antibodies to detect ToxR proteins. The latter were produced by plasmid encoded FLAG-tagged *toxRS*, *toxRS*(Δ264) and *toxR^CC^S* in a *V. cholerae* Δ*toxRS* strain grown in LB medium. Cell extracts were sampled in Laemmli buffer, both with and without the reducing agent ß-mercaptoethanol. In the presence of ß-mercaptoethanol, only a single reduced ToxR or ToxR^CC^ protein band with 35 kDa was observed (data not shown). If cell extracts were not treated with reducing agent, then additional ToxR protein bands became visible. As shown (Fig. 2, lane 4, from bottom to top), ToxR protein bands were visible as an oxidized form at ∼33 kDa and a minor reduced monomer band at 35 kDa, followed by a 70 kDa homodimer and oligomeric forms >170 kDa. In contrast, FLAG-tagged *toxR^CC^S* expression (Fig. 2, lane 5 and 6) only yielded one single monomeric ToxR form migrating at 35 kDa, indicating no existing disulfide bond formation. Interestingly, it was observed that by expressing FLAG-tagged *toxRS*(Δ264), the formation of ToxR disulfide bond dependent homodimer and oligomers was enhanced (Fig. 2, compare lane 1, 2 with lane 3, 4). This observation was also shown in *E. coli* (see below) and demonstrates that *toxRS* coexpression negatively influences the formation of cysteinyl dependent ToxR homodimers and oligomers.

**Figure 3:**
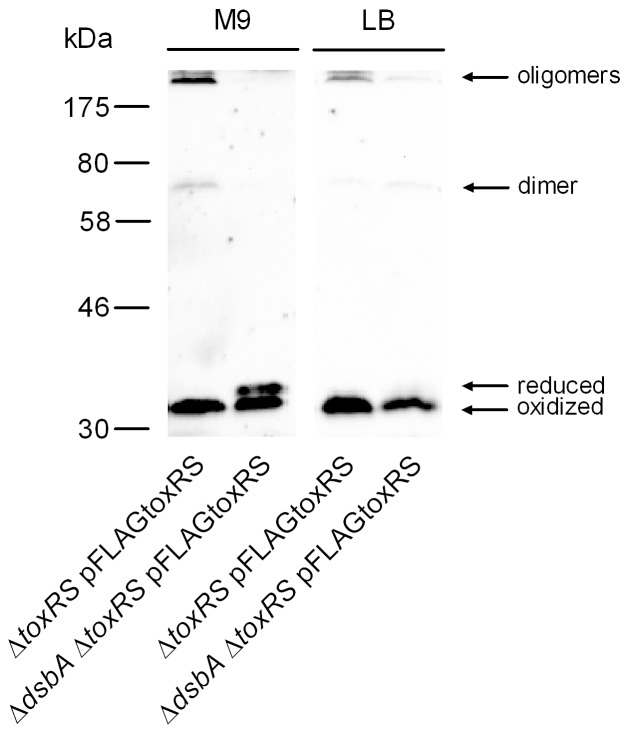
*dsbA* knockout mutant and ToxR forms. Immunoblot analyses are shown using anti-FLAG antibodies to detect FLAG-tagged ToxR produced in *V. cholerae* P27459-S Δ*toxRS* and Δ*toxRS* Δ*dsbA* mutant strain (as indicated in the figure). Bacterial cultures harboring pFLAGtoxRS were grown to mid-log phase in M9 glycerol and in LB broth and induced with IPTG. ToxR mobility in the different samples was monitored and differences for intrachain disulfide bond formation were detected. Immunoblot analysis was performed at least three times, and results were reproducible.

ToxR disulfide bond formation of plasmid encoded FLAG-tagged *toxRS* was monitored in *V. cholerae* Δ*toxRS* and *dsbA^+/−^* strain backgrounds. Cells were grown under two different conditions, M9 glycerol and LB broth. As shown (Fig. 3), if FLAG-tagged *toxRS* was expressed in a *dsbA^+^* background, mainly oxidized ToxR and oligomeric forms were visible, regardless of the growth medium. In contrast, expression of FLAG-tagged *toxRS* in a *dsbA* strain in M9 glycerol medium resulted in both ToxR monomer forms and no observable oligomers, while in LB broth, the oxidized monomer was the dominant expressed ToxR form followed by very minor expressed oligomer forms. Thus, these data demonstrate that disulfide bond formation of ToxR differs in a *dsbA* dependent manner. Importantly, ToxR intrachain disulfide bond formation existed independently of DsbA under LB broth growth conditions, but responded to DsbA function under M9 glycerol growth conditions (Fig. 3). These results indicate a correlation between the activity of DsbA, ToxR intrachain disulfide bond formation and ToxR activity.

### *toxR^CC^* mutant affects porin production

**Figure 4:**
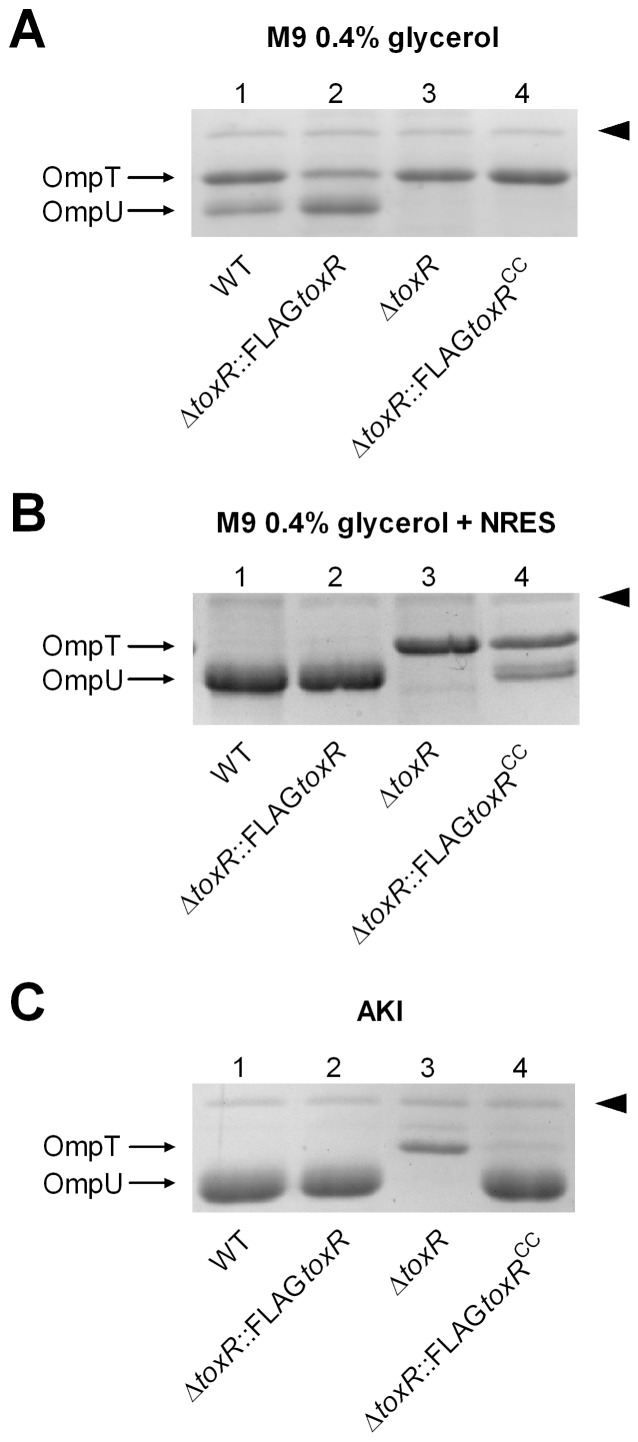
Chromosomal expression of FLAG*toxR* and FLAG*toxR^CC^* and porin regulation. Panel A, B and C, shown are OMP profiles derived from OM preparations, representing WT, Δ*toxR*::FLAG*toxR*, Δ*toxR* and Δ*toxR*::FLAG*toxR^CC^* strains, grown to stationary phase in M9 glycerol (A), M9 glycerol NRES (B) and AKI (C) during the anaerobic growth phase, respectively. Arrows mark OmpU and OmpT. Arrowheads on the right indicate a ToxR independent protein band used as loading control.

**Figure 5:**
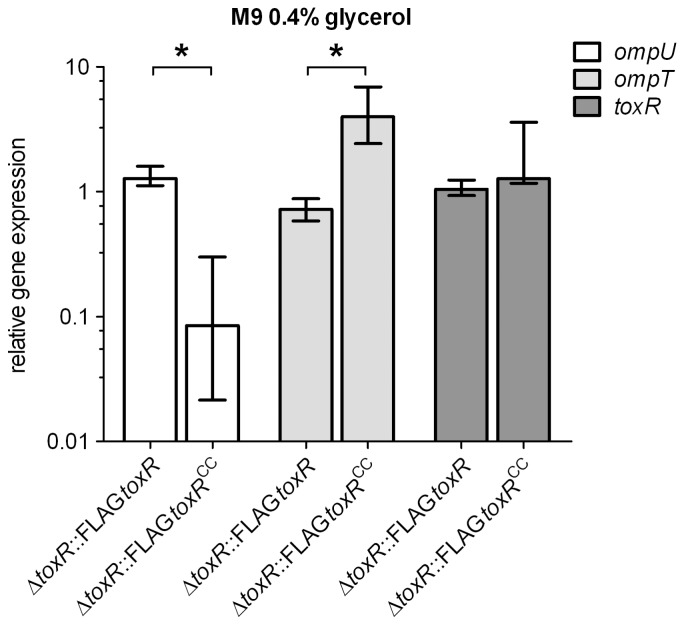
Transcriptional analysis of *toxR* and porin genes *ompU* and *ompT* in *V. cholerae* P27459-S. Using qRT-PCR analysis, transcriptional activity of chromosomal encoding FLAG-tagged *toxR* and *toxR^CC^* strains was monitored for the porin genes *ompU* and *ompT* and also for *toxR* and *toxR^CC^*. mRNA levels of *rpoB* (used as a reference gene) were determined and correlated with the mRNA level of the genes of interest. Data are presented as median fold change and the error bars indicate the interquartile range of each data set. Experiments were performed with six independent samples, the Mann-Whitney U test was used, *P*<0.05.

As shown above, a *dsbA* knockout mutation interfered with disulfide bond formation of ToxR and furthermore resulted in a loss of ToxR activity. Therefore, *toxR^CC^* expression should cause a similar defect in ToxR activity. ToxR regulation is considered non-physiological if plasmid encoded *toxR* is used, because elevated ToxR protein levels counteract ToxR regulation sensitivity [21]. Accordingly, no difference in OmpU and OmpT production was observed by analyzing the OMP profile of Δ*toxRS* strains expressing plasmid encoded FLAG-tagged *toxRS* or *toxR^CC^S* (data not shown). Therefore, single copy gene number and chromosomally expressed *toxR* and *toxR^CC^* were tested under different growth conditions. FLAG-tagged *toxR* and *toxR^CC^* gene alleles were transferred into the chromosomal *toxR* expression locus of a *V. cholerae* Δ*toxR* strain (see Material and Methods). Subsequently, chromosomally produced FLAG-tagged ToxR proteins were collected from membrane extracts derived from cells grown to stationary phase in LB broth and monitored by immunoblot analysis. It is important to note that ToxR proteins could be detected under these conditions, however, the signal intensity was weak such that monitoring ToxR was only possible under high magnification sensitivity (Fig. S3). No signals were detected for samples treated without ß-mercapthoethanol (data not shown). As shown recently, the production of the porins OmpU and OmpT was significantly changed in *V. cholerae* cells if they were grown in a complex broth medium, such as LB, compared to minimal T medium [57]. It was further shown that the pattern of OMP production observed for cells grown in LB broth could be mimicked by the addition of the amino acids L-asparagine, -arginine, -glutamate and –serine (termed NRES) to minimal growth medium [57]. In order to monitor the effect of the *toxR^CC^* mutant on OMP production, standard growth conditions were tested that generally affect porin [57] and virulence factor production, i.e., AKI growth medium [42]. When cells were grown in AKI, M9 glycerol or M9 glycerol NRES medium, the pattern of OmpU and OmpT production in a FLAG-tagged *toxR* strain was similar to that observed for WT (Fig. 4A, B, C, lane 1 and 2, respectively). In contrast and shown by others [25], no OmpU and derepressed OmpT was observed for the Δ*toxR* mutant strain (Fig. 4A, B, C, lane 3). Interestingly, the *toxR^CC^* mutant cells, grown in M9 glycerol medium (Fig. 4A, lane 4), showed no OmpU and derepressed OmpT, similarly as observed for the *dsbAB* mutations (Fig. 1A). Such an effect was also observed in the classical strain O395 (Fig. S2B). Furthermore, production of OmpU was only partially restored and did not reach WT levels in *toxR^CC^* mutant cells grown in M9 NRES medium (Fig. 4B, lane 4). Interestingly, if the *dsbA* mutant strain was grown in M9 glycerol with NRES, OmpU production was similar to that observed for NRES activated WT cells (data not shown). Considering the recently published effects of NRES [57], these data indicate that *dsbA* mutation does not disrupt NRES activated OmpU production, but *toxR^CC^* mutation does. To quantify the effects of *toxR^CC^* on transcription, *ompU*, *ompT* and *toxR* mRNA levels were determined by qRT-PCR analysis of cells grown in M9 glycerol medium for 24 h. The results showed that *toxR* gene transcript levels of FLAG-tagged *toxR* compared to *toxR^CC^* were similar (Fig. 5). In contrast, *ompU* transcription showed a 15-fold reduction in a FLAG-tagged *toxR^CC^* mutant compared to the FLAG-tagged *toxR* strain, whereas *ompT* transcription was 6-fold increased. Thereby, the transcriptional pattern corresponds to the observed porin production profile and is related to a similar transcriptional pattern seen in a *dsbA* mutant (Fig. 1B). It is important to note that in AKI grown WT, FLAG-tagged *toxR* or FLAG-tagged *toxR^CC^* cells, OmpU levels were strongly upregulated, as shown in Fig. 4C. To quantify this observation we monitored *toxR* and *ompU* transcription in WT, using qRT-PCR, by comparing cells grown in M9 glycerol and AKI medium. The obtained results showed a 4-fold upregulation for *ompU* transcription, but no difference in *toxR* transcription (Fig. S4). Therefore, the increase in OmpU production did not correlate with elevated *toxR* transcription.

### ToxR operators negatively influence ToxRS heterodimerization

**Figure 6:**
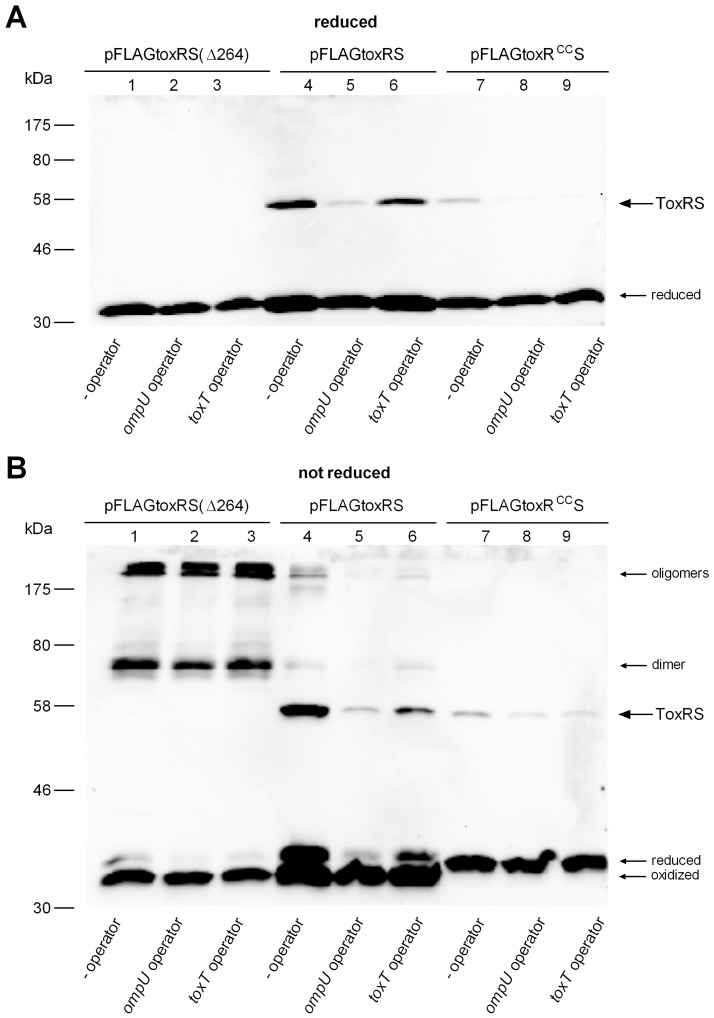
*toxRS* coexpression in *E. coli* XL1-Blue strain. Shown are immunoblot analyses utilizing anti-FLAG antibodies to monitor FLAG-tagged ToxR production of pFLAGtoxRS constructs, performed under reducing (panel A) and non-reducing conditions (panel B). pFLAGtoxRS was expressed in *E. coli* cells grown in LB broth to mid-log phase (OD_600_ of 0.5) and subsequently induced with IPTG for 1 h. From left to right, shown are pFLAGtoxRS(Δ264), pFLAGtoxRS and pFLAGtoxR^CC^S, either containing no ToxR operator sequence or *ompU* or *toxT* operator sequences, respectively. A 55 kDa ToxR cross-reacting protein band, associated with pFLAGtoxRS and pFLAGtoxR^CC^S, is indicated by an arrow. To note, cysteinyl dependent homodimer and oligomer ToxR bands occurred diminished as observed for pFLAGtoxRS in comparison to pFLAGtoxRS(Δ264). Molecular size markers are indicated on the left. Immunoblot analysis was performed at least three times, and results were reproducible.

If pFLAGtoxRS was expressed in *E. coli* cells,grown in LB broth (mid-log phase OD_600_ of 0.5 and subsequently induced with IPTG for 1 h), a novel and stable SDS-resistant FLAG related protein band of about 55 kDa was observed (Fig. 6A, B, lane 4). This size corresponded well with a reported ToxRS heterodimer [34]. This heterodimer is disulfide bond independent, since ToxS does not have any cysteine residues and the heterodimer was also present in SDS-PAGE analysis using samples treated with ß-mercaptoethanol (Fig. 6A, lane 4). To confirm that this protein band represents a ToxRS heterodimer, again pFLAGtoxRS(Δ264) served as negative control, resulting in the loss of the 55 kDa protein band (Fig. 6A, B, lane 1). Thus, these data indicate that this protein band indeed represented a ToxRS heterodimer. Furthermore, as observed in Fig. 6A, B, lane 7, *toxR^CC^S* expression also yielded a heterodimer, which was less pronounced and indicated a decreased interaction of both proteins. Prompted by the observation that ToxRS heterodimer formation only occured in *E. coli* (Fig. 6, lane 4), but not in *V. cholerae* (Fig. 2, lane 1, 2), pFLAGtoxRS plasmids were constructed to additionally contain ToxR operator sites, either of *ompU* [58] or *toxT* [19] (see Material and Methods). Such plasmids were expressed again in *E. coli*, showing that in the presence of ToxR binding sites, ToxRS heterodimer formation appeared strongly diminished (Fig. 6B, lane 4–6) and mainly ToxR monomers with the intrachain disulfide bond were detectable. Furthermore, also a slight decrease in ToxR^CC^S heterodimerization was observed in the presence of ToxR operators (Fig. 6A, B, lane 8, 9). Notably, the presence of ToxR operators had no effect on the appearance of cysteinyl dependent ToxR homodimer or oligomer formation if expressed as *toxRS*(Δ264) (Fig. 6B, lane 1–3). Thus, it seems that ToxR binding sites negatively influenced ToxRS heterodimerization. These data provide a rational explanation for the failure to observe a heterodimer in *V. cholerae*, because numerous ToxR regulated genes [16] and therefore multiple ToxR operators are present in *V. cholerae*.

### Virulence factor production of *toxR^CC^* mutants in El Tor and classical strains and in vivo colonization of mice

In order to characterize a *toxR^CC^* mutant strain for virulence factor production, the strain encoding chromosomally FLAG-tagged *toxR^CC^* was compared with a chromosomally FLAG-tagged *toxR* strain. The levels of CT and TCP were assessed in both biotypes, O1 *V. cholerae* El Tor and O395 classical strain, Table 3. As shown, no significant differences were detectable between FLAG-tagged *toxR^CC^* and *toxR*, while slightly enhanced CT production was observed by comparing FLAG-tagged *toxR* and WT strains. In contrast, a *toxR* deletion strain was about 82-fold reduced for CT production compared to a FLAG-tagged *toxR* O1 El Tor strain and >1,700-fold reduced in the classical strain, Table 3. A *toxS* knockout was about 5- and 3-fold below the CT level compared to the parental strains with FLAG-tagged *toxR* O1 El Tor and classical, respectively see Table 3. Additionally, the CTX-kmΦ transduction frequency was determined for both biotypes, Table 3. This assay relies on the production level of the type IV bundle forming pili TCP and subsequent CTX-kmΦ transduction utilizing a kanamycin encoding CTX-kmΦ [52]. CTX-kmΦ transduction frequencies of WT, FLAG-tagged *toxR* and *toxR^CC^* strains did not show significant differences and for Δ*toxR* mutants, no detectable CTX-kmΦ transductants were observed. Δ*toxS* mutants of O1 El Tor and classical strains compared to a corresponding FLAG-tagged *toxR* strain showed 16- and 7-fold lower frequencies, respectively. In addition, in vivo and in vitro competition assays were performed to further elucidate a putative loss of function for the *toxR^CC^* mutant. A 1∶1 mixture of chromosomally encoded FLAG-tagged *toxR* (LacZ^−^) and FLAG-tagged *toxR^CC^* (LacZ^+^) strains was administered orally to infant mice (in vivo). As a control, LB broth was inoculated with this mixture and incubated for 24 h at 37°C (in vitro). Competitive indices from at least four independent competition experiments were obtained, Table 4, demonstrating no significant difference in colonization fitness between both strains tested. Finally, and as shown above, OMP analysis of the FLAG-tagged *toxR^CC^* mutant strain grown in AKI medium showed a similar porin pattern as obtained for a WT strain (Fig. 4C). Thus, under AKI growth conditions, FLAG-tagged *toxR^CC^* did not disrupt the porin production pattern. In summary, expression of *toxR^CC^* has no effect on virulence gene transcription under the conditions tested. Consequently, these data suggest that other ToxR activation mechanisms exist that do not require ToxR disulfide bond formation.

**Table 3:** Virulence factor production of chromosomal encoded FLAG-tagged *toxR* and FLAG-tagged *toxR^CC^* mutants.

Strain	CTX-kmΦ transduction rate [cfu×ml^−1^]^a^	CT production [ng×ml^−1^]^b^
El Tor P27459-S		
Δ*toxR*::FLAG*toxR*	4.76×10^−5^ (1.5×10^−5^–1.03×10^−4^)	7502 (4341–15734)
Δ*toxR*	<LOD	92 * (77–127)
Δ*toxR*::FLAG*toxR^CC^*	3.83×10^−5^ (2.48×10^−5^–6.84×10^−5^)	5012 (2433–11578)
Δ*toxR*::FLAG*toxR* Δ*toxS*	2.96×10^−6^ (8.15×10^−7^–2.76×10^−5^)	1543 * (988–2570)
WT	4.54×10^−5^ (2.85×10^−5^–6.09×10^−5^)	4654 (2605–7806)
Classical O395		
Δ*toxR*::FLAG*toxR*	1.55×10^−4^ (2.78×10^−5^–1.98×10^−4^)	17178.5 (10372–40044)
Δ*toxR*	<LOD	10 * (10–10)
Δ*toxR*::FLAG*toxR^CC^*	5.71×10^−5^ (1.63×10^−5^–3.13×10^−4^)	12725.5 (3961–48650)
Δ*toxR*::FLAG*toxR* Δ*toxS*	2.22×10^−5^ (8.87×10^−6^–1.01×10^−4^)	5490 (3042–15710)
WT	1.34×10^−4^ (2.02×10^−5^–5.60×10^−4^)	11354.5 (9978–25108)

a median and interquartile range of at least 7 independent experiments.

b median and interquartile range of 9 independent experiments.

* significant by Kruskal-Wallis test followed by Dunn's test of selected pairs of columns with *P*<0.05.

<LOD below limit of detection of 5×10^−8^.

**Table 4:** In vitro and in vivo competition of chromosomal encoded FLAG-tagged *toxR* versus FLAG-tagged *toxR^CC^* mutant in El Tor P27459-S.

in vitro^a^	in vivo^b^
1.04 (0.76–1.22)	2.94 (1.23–3.52)

a median and interquartile range of 12 independent experiments.

b median and interquartile range of 5 independent experiments.

## Discussion

A previous study [34] reported that ToxR formed a heterodimer with ToxS and also existed as a homodimer and monomer based on inter- and intrachain disulfide bonds of cysteine 236 and 293. Moreover, in vitro analysis using the purified periplasmic domain of ToxR showed that ToxR homodimers exist and rely on cysteine 293 by forming an intermolecular disulfide bond [59]. We revisited these earlier characterizations with our work, because we observed that *dsbAB* mutants affect porin production of OmpU and OmpT. Therefore, we focused our studies on the influence of cysteinyl dependent ToxR forms, ToxR activity, ToxRS interaction and response to the Dsb system. As identified in this study, *dsbA*, *dsbB* deletions and *toxR^CC^* mutants showed altered activity for porin regulation. In *V. cholerae*, *dsbAB* and *dsbCD* homologues exist, encoding functions for disulfide bond formation [60] and correction of protein folding [61] that are located in the periplasm. In *E. coli*, it is known that DsbAB activity is important for disulfide bond formation if cells are grown in minimal medium. This is because in full broth media, such as LB, small organic molecules are present, which act as oxidizing agents on cysteine residues and will therefore also lead to disulfide bond formation [56]. As observed in this study, *dsbA* or *dsbB*, but not *dsbC* deletion mutants affected ToxR dependent porin regulation. As shown for *dsbA* or *dsbB* mutant strains grown under M9 glycerol growth conditions, low OmpU and derepressed OmpT levels were observed, which corresponded to altered *ompU* and *ompT* transcription if compared to the WT strain. Interestingly, in a *dsbA* mutant a statistic significant increase of *ompT* transcription was observed, also the OMP profile exposed a slightly higher expressed OmpT band. However, the latter observation also indicated a higher OmpT expression in *dsb* mutants if compared with *toxR* knockout mutant, thereby it can be excluded that *dsbA* deficient disulfide bond formation of ToxR is the cause of increased OmpT expression. Further investigation is necessary to provide an explanation for this observation. No alteration in porin production was observed in the *dsbC* mutant, which suggests that the DsbCD system does not participate in regulating ToxR activity. However, its participation cannot be entirely excluded since DsbCD activity may have an influence on porin production under conditions not tested in this study. Since the *dsbAB* as well as *toxR^CC^* phenotypes were also detectable in classical strain O395, we conclude that the herein characterized ToxR cysteine requirement is significant for *V. cholerae* strains in general.

To identify whether DsbA disulfide oxidoreductase activity per se is influencing ToxR forms, FLAG-tagged *toxRS* expression was analyzed in a *dsbA toxRS* knockout strain. Interestingly, it was demonstrated that by expression of *toxRS* in the absence of *dsbA* ToxR showed intrachain disufide bond and reduced monomer forms in M9 glycerol, but only the intrachain disulfide bond form in LB broth. The latter is explainable because small organic molecules are present in LB and can catalyze intrachain disulfide bond formation supporting ToxR activity. Taken together, these data imply that ToxR activity is stimulated by DsbA or alternative oxidizing mechanisms, producing thiol-dependent intrachain disulfide bond formation.

To confirm *dsbA* dependent ToxR phenotypes, a *toxR^CC^* mutant was characterized, which is defined by amino acid substitutions of cysteines to serines. The appearance and electrophoretic mobility of ToxR^CC^ tested without reducing agents in SDS-PAGE was as expected. ToxR^CC^ showed no intrachain disulfide bonded monomer, homodimer or oligomeric forms. Instead, ToxR^CC^ only produceed a single protein band corresponding to the size of a reduced ToxR monomer. Therefore, the activity of ToxR^CC^ was associated with the reduced monomeric form. By using a chromosomally encoded *toxR^CC^* mutant, it was demonstrated that porin production is influenced profoundly, quite similar to that observed for the *dsbA* or *dsbB* deletion mutants. Additionally, we tested a double mutant comprising *dsbA* and *toxR^CC^* and observed no differences in the OMP profile compared to a *toxR^CC^* mutant (data not shown). Furthermore, no difference was observed between *toxR* or *toxR^CC^* transcription levels, hence we argue that *toxR^CC^* represents a mutation affecting ToxR activity. Therefore, we provide evidence that DsbA activity is targeting cysteine residues of ToxR and this influences ToxR activation.

As shown recently [57], when an O1 El Tor *V. cholerae* strain was grown in minimal medium OmpT was expressed as a major porin and no OmpU protein was observed. In LB broth, the porin production pattern was reversed. If NRES amino acids were added to the minimal medium, the porin production profile appeared similar to that observed for cells cultured in LB broth. Mey et al. further showed that NRES amino acids added to the minimal medium led to elevated *toxR* transcription, which they concluded, is the cause for the switched porin production. As shown in here, we also confirmed the NRES effect in showing that addition of NRES to M9 glycerol medium enhances OmpU production for WT and FLAG-tagged *toxR* strains. Also FLAG-tagged *toxR^CC^* mutant cells responded to NRES but to a lower extend. However, *toxR^CC^* mutant cells grown in AKI showed maximum OmpU production, similar as observed for FLAG-tagged *toxR* or WT strains. Hence, we assumed that under AKI growth conditions, elevated *toxR* and *toxR^CC^* transcription would occur, explaining the increase in OmpU production. This assumption was not confirmed, since qRT-PCR analysis of WT cells cultured in M9 glycerol compared with AKI cultures showed no significant difference in *toxR* transcription. These data indicate, that other mechanisms exist, which influence ToxR activity. For example, we cannot rule out, that DsbAB activity influences *ompU* post-transcriptional, -translational or secretion pathways, neither can we exclude that *dsbA* or *toxR^CC^* mutants are solely responsible for the observed decreased *ompU* transcription. Therefore, other yet unknown factors may contribute or facilitate OmpU expression, especially under growth conditions such as AKI or LB broth media. Also to mention is that if *toxR^CC^* is expressed from multi-copy plasmid, then we observed that cells were producing high OmpU levels. Similar behavior of ToxR activity was observed earlier [21], however such conditions were regarded as non-physiological, hence we will not address this for further discussions. In summary, our data consistently show that under minimal growth conditions, porin regulation solely depends on ToxR intrachain disulfide bond formation if *toxR* is expressed from its chromosomal loci.

If *toxS* was coexpressed along with *toxR*, cysteinyl dependent ToxR homodimer and oligomer formation was decreased as shown in our results. The latter finding is supported by earlier published data [34], showing suppression of the ToxR homodimer in the presence of ToxS. According to these findings, it can be assumed that under physiological expression conditions, ToxRS counteract the formation of homodimers. We further suggest, that oligomer ToxR forms most likely represent artifacts, resulting from *toxR* overexpression, since this form was not observed earlier, if chromosomal expressed *toxR* was monitored [34]. As found earlier by others, e.g., [21], [39] the combined action of ToxR and ToxS contributed to full ToxR operational activity and both proteins were shown to form a heterodimer [34]. With the expression system we used, heterodimer formation was observed only in *E. coli*, it was SDS- and heat-resistant and was stably detectable without any cross-linking chemistry. This finding is not exceptional, since other SDS-resistant protein-protein interactions are known to occur [62]–[64]. Such interactions were explained by the failure of SDS to access tightly formed contact sites between the proteins. In contrast, a ToxRS heterodimer could not be visualized if both genes were coexpressed in *V. cholerae*. To further characterize this, defined ToxR binding sites, located upstream of *ompU* [58] and *toxT* [19], were subcloned onto the *toxRS* expression plasmids and analyzed in *E. coli*. Interestingly, if ToxR binding sites were present, heterodimer signals were strongly diminished, indicating that the presence of operator sites negatively interfered with ToxRS heterodimerization. Earlier data also described ToxRS heterodimerization in *V. cholerae*, using cross-linking chemistry [34]. There, detection of the heterodimer was very weak. Since *V. cholerae* contained multiple ToxR regulated genes [16], the presented data may support the view whereby only weak ToxRS heterodimerization might be observable in the presence of ToxR operator sites. Interestingly, in another report [39] it was shown that ToxS was not necessary for ToxR binding to DNA in vitro. Therefore, it seems questionable whether ToxRS heterodimers can be found in a DNA bound state. So far it can only be speculated whether disaggregation of heterodimer is a consequence of ToxR binding and this needs further characterization.

Finally, we tested virulence factor production in O1 El Tor or classical strains and neither the level of CT, nor CTX-kmΦ transduction frequencies showed any significant differences between FLAG-tagged *toxR^CC^* and *toxR* strains. This indicated that under virulence factor inducing conditions, ToxR^CC^ can participate in the regulation cascade of the virulence factor production system. Moreover, the *toxR^CC^* mutant in O1 El Tor *V. cholerae* strain did also not display a phenotype for in vivo colonization. In summary, this suggests that cysteine associated ToxR forms are dispensable for ToxR activity under virulence factor inducing conditions. Thereby, we argue that multiple ToxRS activation conditions may exist, which do not rely on thiol-dependent disulfide bond forms. So far, we cannot explain why porin gene regulation seems to respond sensitive to ToxR disulfide bond formation, whereas virulence gene expression is not. Recently, a possible hint was provided by Morgan and colleagues [29]. They published data addressing the isolation of *toxR* point mutations, which differentially target *toxT* and *ompU* transcription. All of their *toxR* mutations were found in the cytoplasmic part of ToxR. For example, for amino acid residues V71, F69 and E39 it was proposed that they interfere with RNAP engagement on the *ompU* promoter, rather then with DNA binding. Interestingly, the same point mutants had much less effect on *toxT* activation. In contrast, amino acids R65 and D73 affected more severely *ompU* activation than *toxT*. Based on these results, the authors concluded that the facing of ToxR upon operator binding seems differently oriented for the promoter regions of *ompU* and *toxT*. Thereby, it can only be speculated that ToxR^CC^ protein configuration is sufficient to serve for *toxT* activation by correctly facing to the *toxT* promoter, but becomes conditionally insufficient for *ompU* activation for yet unknown reasons.

Finally, the question arises whether ToxR may represent a thiol-based redox switch regulator. Although, several periplasmic proteins depend on DsbAB folding activity to obtain function, e.g. PhoA [65], while no examples to our knowledge exist for DsbAB depending thiol-based redox switches for periplasmic proteins. However, the latter does not exclude the possibility that defined environmental conditions exist, e.g., in the aquatic environment, that modulate thiol-dependent intrachain disulfide bond formation in ToxR, hence leading to changes in ToxR activity. For example, such influences could be derived from stress responses, as *dsb* gene transcription is under the control of the σ^E^-membrane stress pathway in *V. cholerae* and additionally of the Cxp regulon as shown in *E. coli* [66]–[68]. ToxR activity may also be linked to the cellular metabolism status of the cells. The status may be reflected by electron transfer activity, which is known to influence DsbAB activity whereby menaquinone or ubiquinone act as recipients for e^−^ derived from disulfide bond formations [55].

## Supporting Information

Figure S1 Growth and cell survival for P27459-S and Δ*dsbA* mutant strains. Shown are growth curves (OD_600_ left Y axis) and colony forming units (cfu/ml right Y axis) of WT strain P27459-S and corresponding Δ*dsbA* strains over 72 h in M9 minimal media supplemented with glycerol (0.4%).
(TIF)

Figure S2 OMP profiles of *V. cholerae* O1 classical strain O395. Arrows indicate OmpU and OmpT. Panel A, shown are WT O395, Δ*toxR*, Δ*dsbA* and Δ*dsbB* strains grown to stationary phase in M9 glycerol medium. Panel B, shown are WT O395, Δ*toxR*, Δ*toxR*::FLAG*toxR* and Δ*toxR*::FLAG*toxR^CC^* strains. Cells were grown to stationary phase in LB broth medium.. Arrowheads on the right indicate a ToxR independent protein band used as loading control.
(TIF)

Figure S3 Detection of chromosomal encoded FLAG-tagged ToxR expressed fusion proteins. Immunoblot analysis is shown, using anti-FLAG antibodies to detect chromosomal expression of FLAG-tagged *toxR* and *toxR^CC^* in *V. cholerae* P27459-S and mutant strains Δ*toxR* and Δ*toxRS* of isolated membrane fractions. Cross-reacting background bands are marked with asterisks and ToxR is indicated by an arrow. Molecular size markers are indicated on the left. Immunoblot analysis was performed at least two times, and results were reproducible.
(TIF)

Figure S4 Transcriptional analysis of *toxR* and porin gene *ompU* in *V. cholerae* P27459-S grown in M9 glycerol compared to AKI conditions. The WT strain was cultured in M9 glycerol medium to mid log growth phase and shifted to fresh M9 glycerol or AKI medium for 45 min. Subsequently mRNA was prepared and qRT-PCR was performed for the *ompU* porin gene and also for *toxR*. mRNA level of 16S rRNA was determined as a reference and correlated with the mRNA level of the genes of interest. Experiments were performed with three independent samples and data represent means and standard deviations. The unpaired t test was used, *P*<0.05.
(TIF)
