# Supplementary figures and images for: Disulfide Bond Formation and ToxR Activity in Vibrio cholerae
Source: PLoS One. 2012 Oct 29;7(10):e47756. doi: 10.1371/journal.pone.0047756 (PMC3483227; doi:10.1371/journal.pone.0047756)

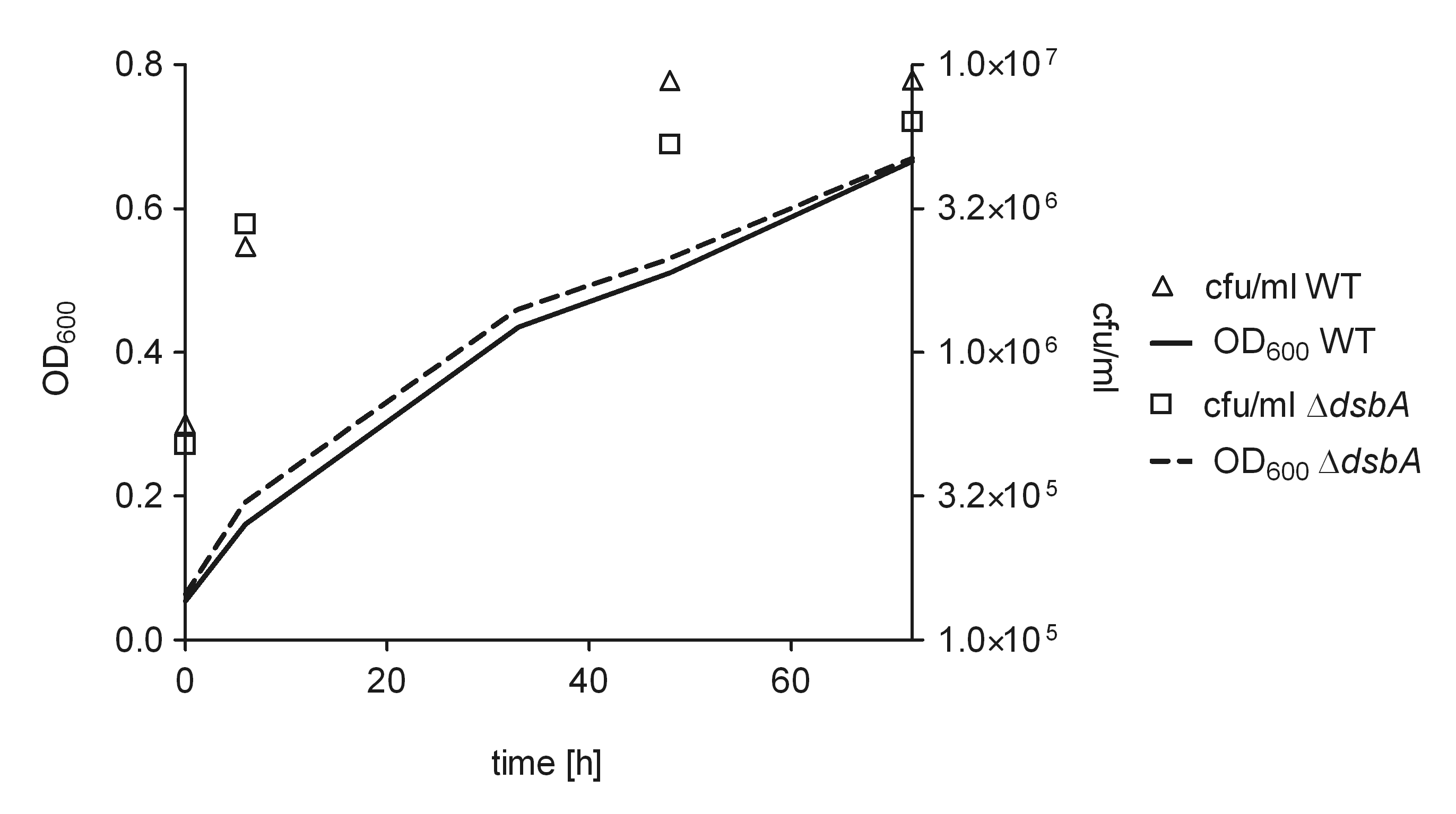

Supplement: Figure S1 — Growth and cell survival for P27459-S and ΔdsbA mutant strains. Shown are growth curves (OD600 left Y axis) and colony forming units (cfu/ml right Y axis) of WT strain P27459-S and corresponding ΔdsbA strains over 72 h in M9 minimal media supplemented with glycerol (0.4%). (TIF) [file pone.0047756.s001.tif]

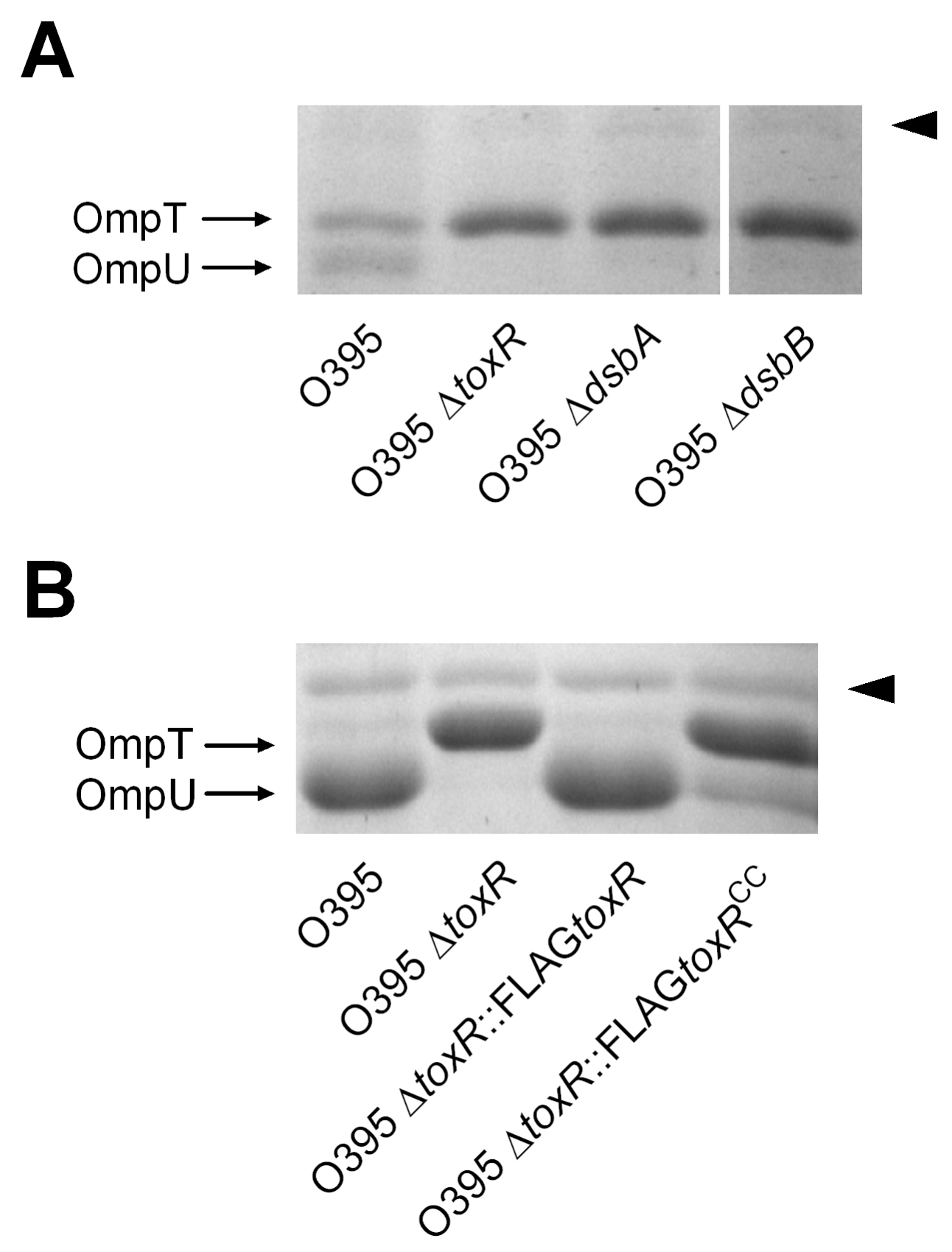

Supplement: Figure S2 — OMP profiles of V. cholerae O1 classical strain O395. Arrows indicate OmpU and OmpT. Panel A, shown are WT O395, ΔtoxR, ΔdsbA and ΔdsbB strains grown to stationary phase in M9 glycerol medium. Panel B, shown are WT O395, ΔtoxR, ΔtoxR::FLAGtoxR and ΔtoxR::FLAGtoxRCC strains. Cells were grown to stationary phase in LB broth medium.. Arrowheads on the right indicate a ToxR independent protein band used as loading control. (TIF) [file pone.0047756.s002.tif]

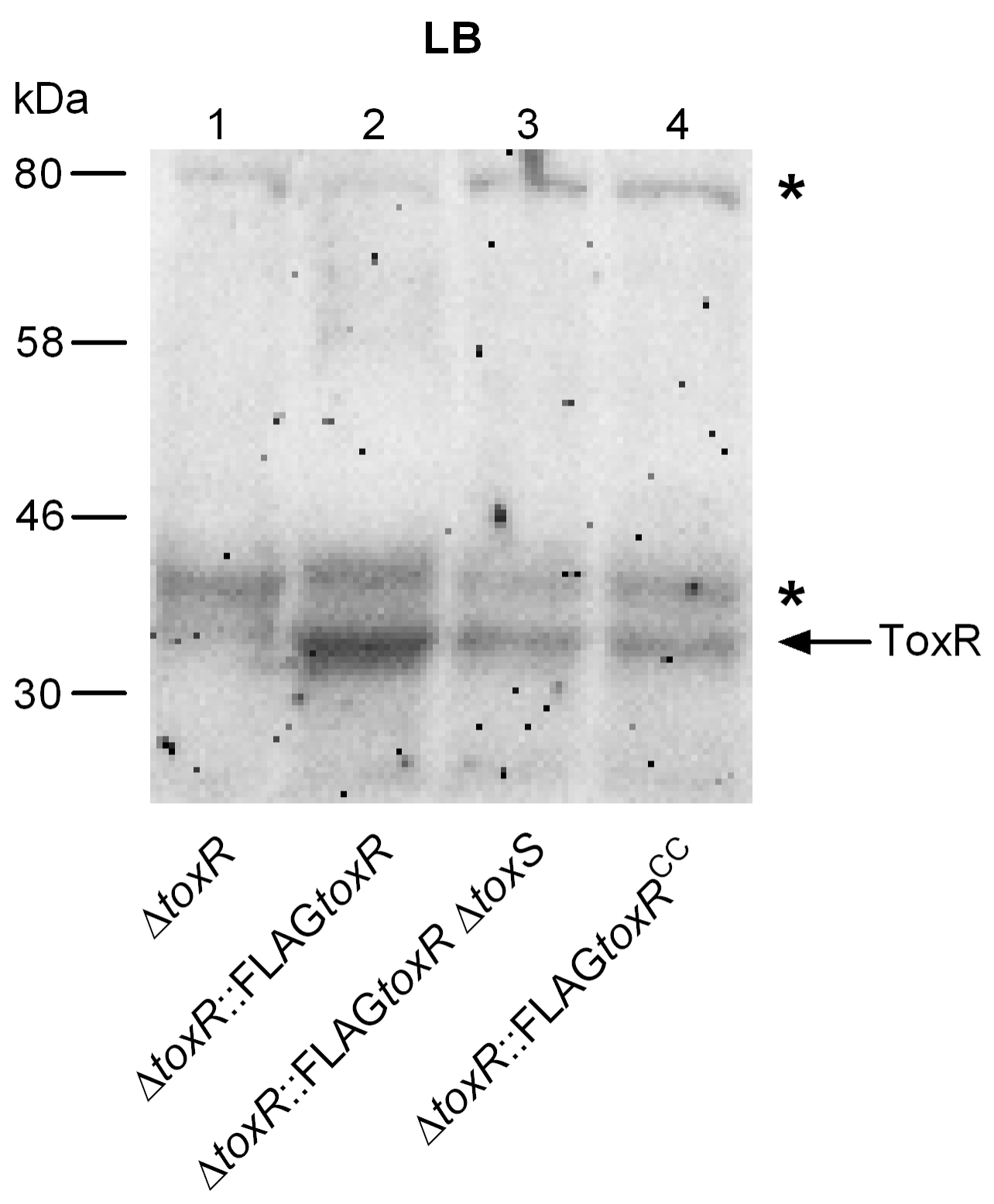

Supplement: Figure S3 — Detection of chromosomal encoded FLAG-tagged ToxR expressed fusion proteins. Immunoblot analysis is shown, using anti-FLAG antibodies to detect chromosomal expression of FLAG-tagged toxR and toxRCC in V. cholerae P27459-S and mutant strains ΔtoxR and ΔtoxRS of isolated membrane fractions. Cross-reacting background bands are marked with asterisks and ToxR is indicated by an arrow. Molecular size markers are indicated on the left. Immunoblot analysis was performed at least two times, and results were reproducible. (TIF) [file pone.0047756.s003.tif]

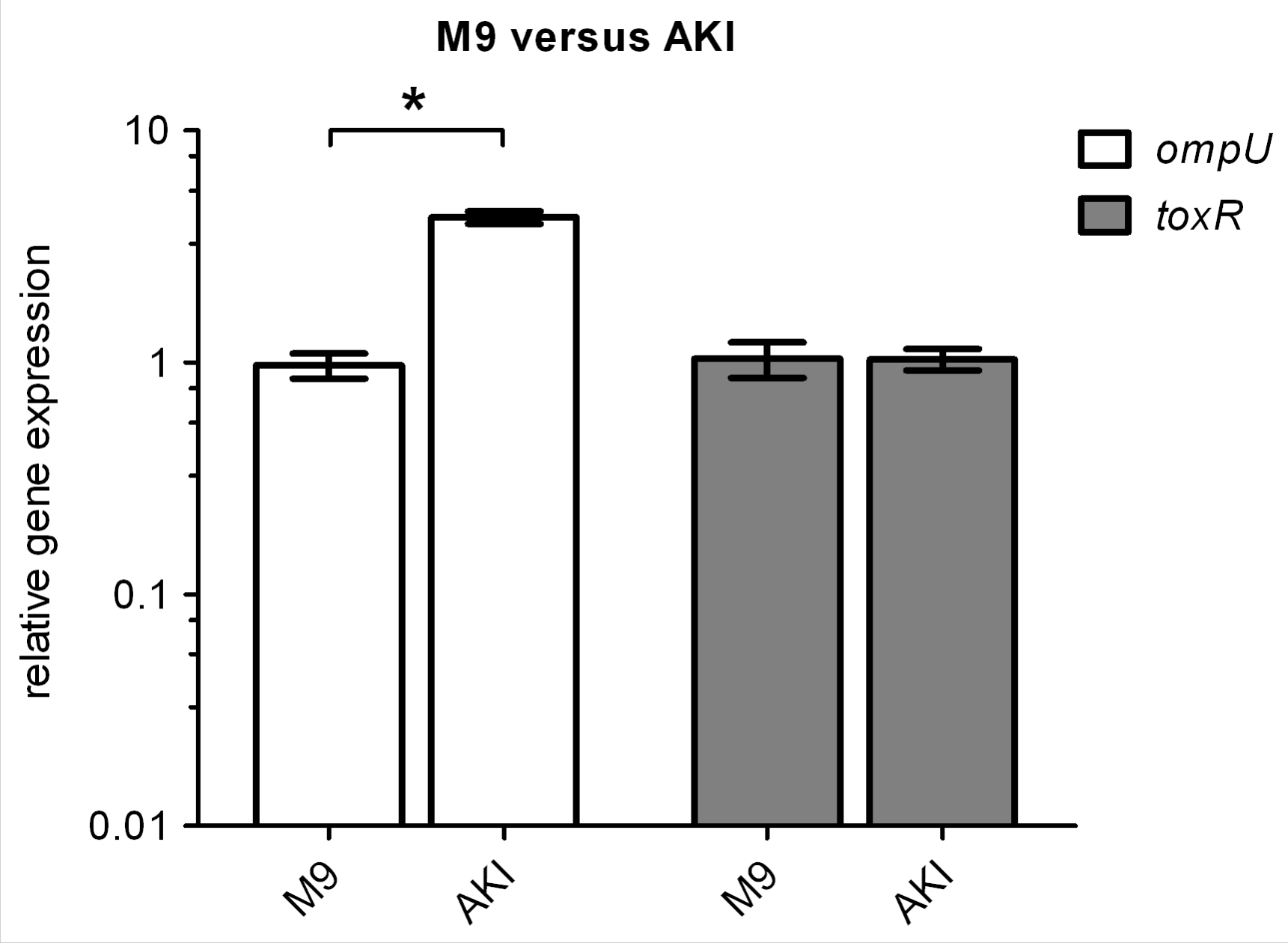

Supplement: Figure S4 — Transcriptional analysis of toxR and porin gene ompU in V. cholerae P27459-S grown in M9 glycerol compared to AKI conditions. The WT strain was cultured in M9 glycerol medium to mid log growth phase and shifted to fresh M9 glycerol or AKI medium for 45 min. Subsequently mRNA was prepared and qRT-PCR was performed for the ompU porin gene and also for toxR. mRNA level of 16S rRNA was determined as a reference and correlated with the mRNA level of the genes of interest. Experiments were performed with three independent samples and data represent means and standard deviations. The unpaired t test was used, P<0.05. (TIF) [file pone.0047756.s004.tif]
